# Effect of chronic prenatal exposure to the food additive titanium dioxide E171 on respiratory activity in newborn mice

**DOI:** 10.3389/fped.2024.1337865

**Published:** 2024-02-29

**Authors:** Eloïse Colnot, Julie O’Reilly, Didier Morin

**Affiliations:** ^1^ CNRS, INCIA, University of Bordeaux, Bordeaux, France; ^2^ Department of Health, Safety and Environment, Bordeaux Institute of Technology, University of Bordeaux, Gradignan, France

**Keywords:** respiration, titanium dioxide nanoparticles, neurotoxicity, maternal exposure, neurodevelopment, newborn mice

## Abstract

Nanoparticles (NPs) possess unique properties that make their use valuable in all industries. Titanium dioxide (TiO_2_) NPs are extensively used as a white pigment in food (labeled under the European number E171) and personal care products, which creates a significant potential for chronic consumer exposure. Concerns about the potential toxic effects of TiO_2_ NPs have arisen, particularly in vulnerable populations, including pregnant women and infants. Recently, human materno-fetal transfer of E171 was demonstrated, and simultaneously, we reported that chronic prenatal exposure to reference P25 TiO_2_ NPs was found to alter the developing respiratory neural networks. In this study, using whole body plethysmography from postnatal day (P) 0 to P7, we assessed the respiratory function of newborn mice born to mothers fed with E171 during pregnancy. We also evaluated the potential alterations to respiratory centers by using brainstem-spinal cord electrophysiological recordings from P0 to P6. Our study reveals that E171-prenatally exposed animals displayed an abnormally elevated breathing rate from P3 onwards. From P5 to P6, the respiratory-related burst frequency generated by the isolated brainstem-spinal cord preparations was significantly higher in E171-exposed animals than in non-exposed animals. These findings demonstrate prenatal toxicity of E171 to the developing respiratory function and may contribute to policy-making regarding the use of TiO_2_ NPs.

## 1 Introduction

One of the most widely used nanoparticle (NP) is titanium dioxide (TiO_2_). Due to its whitening properties, TiO_2_ is extensively employed as a pigment in food products (under the European E number E171), as well as in paints, enamels, plastics, paper, pharmaceuticals and personal care products. The widespread use of TiO_2_ in personal care and food products presents a significant potential for chronic consumer exposure through skin penetrations and ingestion. The European Food Safety Authority (EFSA) estimated typical adult TiO_2_ exposure at 0.3–3.8 mg per kg body weight per day (mg/kgBW/day) in the adult population (1). Remarkably, children are disproportionately exposed to TiO_2_ (0.9–6.9 mg/kgBW/day), primarily due to their higher consumption of TiO_2_-rich sweets. Such widespread exposure has raised concern about potential health hazards associated with TiO_2_ NPs, especially for vulnerable populations, including pregnant women and infants. Recently, the EFSA published an updated safety assessment of E171, in which the panel concluded that TiO_2_ could no longer be considered safe as a food additive (2). The European Commission proposed to member states the removal of E171 from the union list of food additives, and finally, even though the European Union has prohibited the use of the food-grade additive E171 due to concerns about the potential presence of nanoscale TiO_2_, it is still found in stock products and remains permissible in other countries.

In a recent study, human materno-fetal transfer of TiO_2_ NPs was demonstrated by analyzing placentae and meconium (3). The underdeveloped defense mechanisms in fetuses and infants make them more vulnerable to toxic substances, and accumulating evidence suggests fetotoxicity from TiO_2_ NPs. In mice, maternal exposure to TiO_2_ NPs through oral administration impairs placental development and inhibits the formation of fetal vessel (4). TiO_2_ NPs were also found to cross the placental barrier and directly interfere with mouse fetal development (5). Consequently, TiO_2_ NPs may impact the central nervous system during its developmental period when it is most vulnerable (6). Indeed, maternal exposure to TiO_2_ NPs was reported to induce learning and memory impairments in rat offspring (7) and behavioral deficits relevant to autism spectrum disorder in mouse offspring (8). However, few studies have examined the effects of TiO_2_ NP exposure on the developing neural networks responsible for essential motor functions, such as respiration.

The primary function of respiration is to supply oxygen to the cells to carry out their metabolic functions. The respiratory system consists of a peripheral apparatus controlled by neural populations in the brainstem. Two brainstem neural networks play a crucial role in generating the respiratory rhythm: the parafacial respiratory group (pFRG), which controls expiratory activity, and the preBötzinger complex (PBC), which controls inspiratory drive (9). The pFRG and the PBC emerge in mice at embryonic day (E) 14.5 and E15.5, respectively, to form a precursor of the neonatal respiratory rhythm generator, which is already functional during the last third of gestation (10) and plays a crucial role in the development of respiratory motoneurons and muscles. Recently, using the reference P25 TiO_2_ NPs, we reported that a maternal exposure to these particles during pregnancy affected the normal development and operation of the respiratory centers in progeny (11). However, the consumer exposure from P25 TiO_2_ NPs is relatively limited compared to the TiO_2_ particles contained in the food additive E171. Due to the unique physicochemical properties of E171 and P25, the potential adverse effects of E171 on developing respiratory centers have remained unexplored.

The present study was undertaken to contribute further to the discussion on the appropriate regulatory measures to be adopted regarding E171 to ensure consumer protection. The study aimed to investigate the potential toxicity of chronic prenatal exposure to E171 on the activity of the respiratory centers of newborn mice. E171 was administered chronically during pregnancy through voluntary oral ingestion. The respiratory function of newborn mice was assessed using *in vivo* whole-body plethysmography. To determine any potential impact of E171 on the central respiratory network, spontaneous inspiratory-related motor output was recorded from the cervical C4 nerve roots in brainstem-spinal cord preparations.

## 2 Materials and methods

### 2.1 Chemicals

E171 particles were kindly provided by Marie-Hélène Ropers (INRA Unity BIA, Nantes, France). Their process for recovery of E171 particles from commercial food and subsequent particle characterization has been described (12, 13). Briefly, commercial sweets covered with a TiO_2_-containing coating were selected, and the coating was isolated after dispersion in deionized water. TiO_2_ particles were then separated from organic molecules by water-washing. Size, crystalline phase (anatase, rutile) and zeta potential (surface charge) of recovered E171 particles were characterized by transmission electron microscopy, x-ray diffraction and laser Doppler electrophoresis. The mean individual particle size was 115 ± 31 nm with a nanoparticular fraction of 36%. It was composed essentially of anatase and the zeta potential was negative at biological pH (12). E171 stock solutions were prepared by dispersing the white powder in distilled water.

### 2.2 Animals

Experiments were performed on mice belonging to the OF1 strain. Mice were bred and raised under standard housing conditions (12:12 h light-dark cycle, food and water *ad libitum*, room temperature 24°C) in the laboratory animal facility. Animal procedures were conducted in accordance with the provisions for animal care and use described in the European Communities Council directive of 2010 (2010/63/EU) and French law (87/848). All experimental procedures were also conducted in accordance with the guidelines of the local Ethics of Animal Experiments committee of the University of Bordeaux (Permit number: 20140).

On the day of proestrus, females were housed overnight with sexually mature males and vaginal plugs were checked the following day. The occurrence of vaginal plug was designated as embryonic day (E) 0. Pregnant females were individually placed in maternity cages.

### 2.3 Chronic exposure to E171 during pregnancy

Pregnant mice were divided into 2 groups: a sham group and an exposed group to E171 at a dose of 600 µg/g body weight. To select the dose of E171 used, we based our decision on two factors: (i) the highest dose used for the reference P25 (composed of 100% TiO_2_ NPs) in our previous study (11), and (ii) the proportion of TiO_2_ NPs present in E171 [which is 36% (12, 13), three times less than in P25]. In order to maintain consistency in the quantity of TiO_2_ NPs across both studies, we selected a dose of E171 (600 µg/g) three times higher than that employed for P25.

The European Food Safety Authority calculated that a human adult is usually exposed to 0.3–3.8 mg of TiO_2_ per kilogram of body weight per day (1). We then estimated that the total amount of TiO_2_ NPs (36% in E171) administered to a pregnant mouse over the 19-day gestation was approximately 500-fold less than the estimated quantity taken in by a woman during the duration of her pregnancy (280 days).

E171 was administered by voluntary oral ingestion daily from E0 until delivery. Each day prior the start of the active period, pregnant mice, which were isolated in individual cage, were weighed, and a feeding dish was prepared by mixing E171 into chocolate spread (Nutella, Ferrero, Alba, Italy). The chocolate spread (0.5 mg/g body weight) was selected for its consistency and palatability and was confirmed to be free of E171. The sham group only received chocolate spread only. After each exposure, we visually controlled that the feeder had been emptied, thus confirming that the chocolate mixture had been completely ingested by the animal. This voluntary ingestion method was used to minimize the stress on the dams and to more closely resemble human exposure.

### 2.4 Whole-body plethysmography

Whole body plethysmography was performed using methods previously described (11). Briefly, respiratory activity in conscious, unrestrained neonatal mice (with at least 2 litters per group) was assessed by whole body plethysmography at birth and daily until P7. After determining their weight and craniocaudal length, each pup was placed in a custom 50 ml barometric chamber. The chamber was positioned under a heating lamp to prevent heat loss, and an air conditioning system was utilized to maintain the room temperature at 24 ± 1°C.

Breathing was continuously recorded for at least 5 min. Dynamic pressure changes in the plethysmography chamber were transduced using a differential pressure transducer (emka Technologies, Paris, France), with reference to the laboratory room. The signal was amplified through an AMP amplifier (emka Technologies), digitized (sampling rate: 1 kHz), acquired using iox2 software (emka Technologies), and then exported to Spike2 (Cambridge Electronic Design, Cambridge, UK) for analysis. The vertical movement of the tracing represents a measure of the dynamic pressure change, with inspiration being recorded as a downward deflection.

Before each set of measurements, a volume of 10 ml of air was flushed into the empty chamber using a syringe for calibration. After plethysmography, the subjects were returned to their home cages for 24 h.

### 2.5 Breathing data analysis

A bout of continuous quiet resting breathing (i.e., without significant limb, body, and head movements) was analyzed using Spike2 (Cambridge Electronic Design, Cambridge, UK). The instantaneous respiratory cycle period (Pinst) was determined for each cycle, measured from the onset of one inspiration to the next. All identified breaths were manually verified for accuracy. The breathing rate was calculated as the reciprocal of the average Pinst. Respiratory pauses were defined as the absence of breathing movements lasting longer than 3 times the median Pinst. The duration and frequency (number per unit of time) of these pauses were measured.

### 2.6 Brainstem-spinal cord preparation

Brainstem-spinal cord preparation was performed using methods previously described (14). This preparation includes all the central respiratory network elements required to spontaneously generate respiratory-related motor activity for extended periods. Briefly, neonatal mice aged P0–P6 were deeply anesthetized with isoflurane (4%) until they showed no reflex response to tail pinching. The animals were then promptly decerebrated, transected below the shoulders, and placed in ice-chilled (4°C) artificial cerebrospinal fluid (aCSF) containing the following concentrations (in mM): 125 NaCl, 3.35 KCl, 0.58 NaH2PO4, 1.26 CaCl2, 1.15 MgCl2, 21 NaHCO3, and 30 D-glucose (Acros Organics, Fair Lawn, NJ, USA). The pH was adjusted to 7.4 with NaOH, and the solution was saturated with 95% O_2_ and 5% CO_2_. Under a stereomicroscope (Model Stemi 2000, Carl Zeiss, Jena, Germany), the skin, muscles, braincase, vertebrae, and cerebellum were removed. The brainstem was transected rostrally at the caudal cerebellar artery, corresponding to the rostral pole of the facial nucleus.

The preparations were continually perfused with oxygenated aCSF (95% O_2_ and 5% CO_2_) using a peristaltic pump (Model Minipuls 3, Gilson, Middleton, WI, USA) at a flow rate of 10 ml/min. The temperature of the perfusate was maintained between 22.5°C and 24.5°C.

### 2.7 Extracellular recording

Spontaneous inspiratory-related motor discharge was recorded from the proximal cut end of cervical C4 ventral roots, which contain axons of phrenic motor neurons that innervate the diaphragm. Glass suction electrodes were pulled from 1.5 mm diameter borosilicate glass capillaries using a vertical micropipette puller (Model PP-83, Narishige, Tokyo, Japan). The electrode tip diameter was matched to that of the ventral root using a diamond knife, filled with aCSF, and applied to the root. C4 activity was continuously recorded online for at least 30 min. Signals were amplified (x10,000) and band-pass filtered (0.1–1 kHz) using a differential AC amplifier (A-M Systems, Carlsborg, WA, USA). The signal was digitized (sampling rate: 2 kHz), acquired via a CED 1401 interface, and stored on a computer using Spike2 software (Cambridge Electronic Design, Cambridge, UK).

Cycle duration was measured from the onset of one burst to the onset of the next. Instantaneous frequency was calculated as the reciprocal of the cycle duration, averaged, and expressed as bursts per minute.

### 2.8 Statistical analyses

Variables were assessed for normality and homogeneity of variances to determine whether parametric or non-parametric statistical tests should be employed. Statistical analyses were conducted using SigmaPlot 11.0 (Systat), with values expressed as mean ± SEM. A Student's *t*-test or Mann–Whitney test (for non-normally distributed data) was employed to compare the means of two groups. Statistical significance was considered when the *p*-value was < 0.05.

## 3 Results

### 3.1 Exposure to E171 does not impact gestational and morphological characteristics

**Figure 1:**
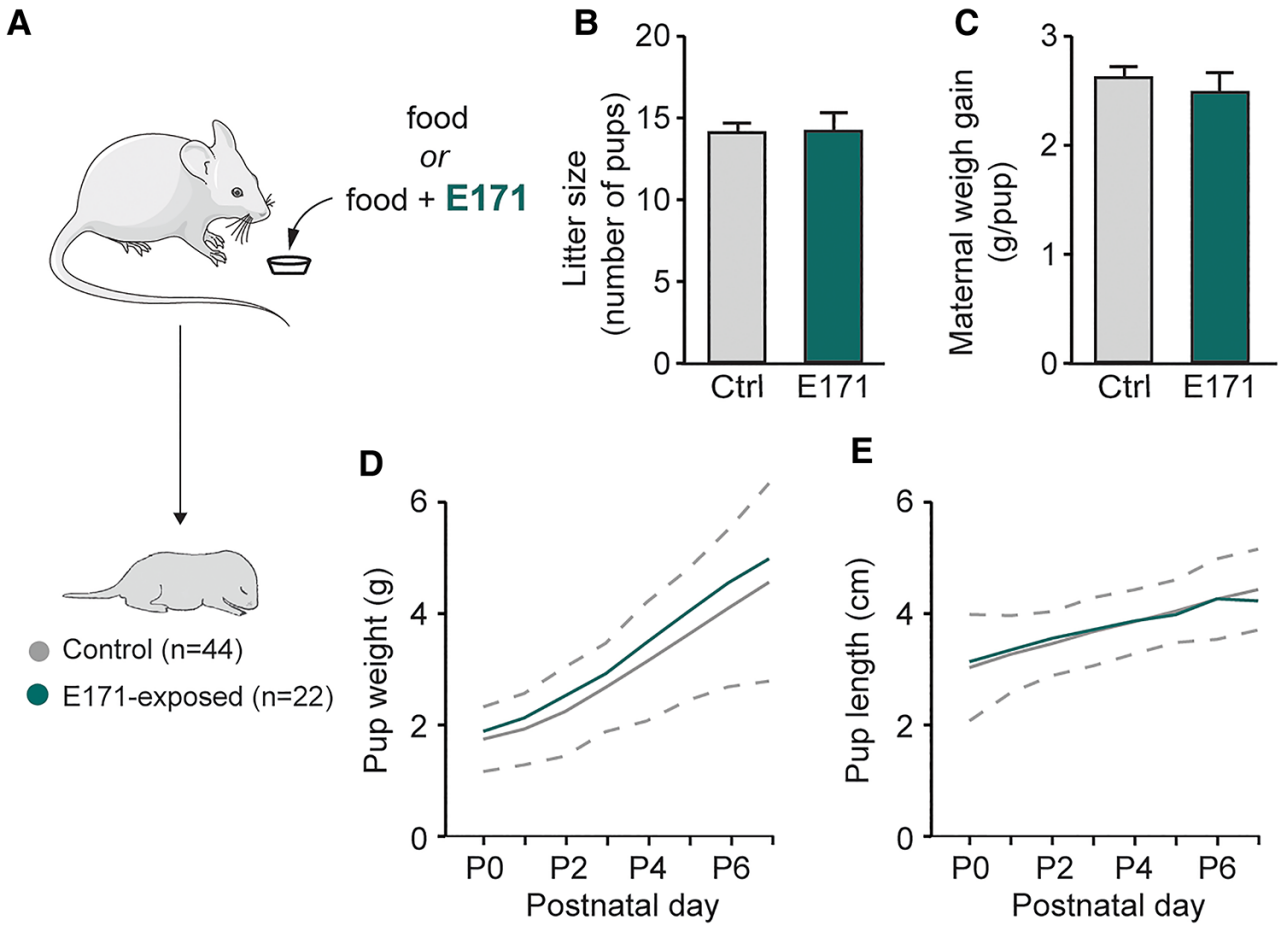
Weight and size of pregnant mice and offspring in non-exposed and prenatally E171-exposed groups. (**A**) Schematics of experimental procedure. (**B**,**C**) Bar charts (mean ± SEM) illustrating litter size (**B**) and maternal weight gain per neonate (**C**) in non-exposed control (Ctrl, grey bars) and prenatally E171 NP-exposed (green bars) groups. (**D**,**E**) Scatter plots showing changes in weight (**D**) and size (**E**) of the neonates during the first postnatal week under these two experimental conditions. Area, which is delimited by ± 2 SEM (dotted line) of the mean of non-exposed animals, represents normal postnatal growth. The mouse image is from Servier Medical Art website (smart.servier.com). ns, not statistically significant.

In order to study potential alterations resulting from prenatal exposure to E171 at the end of gestation, we initially measured gestational and morphological parameters of pregnant mice and newborns (Figure 1A). During gestation, the weight gain of the mice was monitored, and the number of offspring per litter was recorded at birth. The analysis showed that E171 exposure did not affect either the litter size (14 ± 0.8 offspring in the control group (*n* = 16) vs. 14 ± 1.1 offspring in the group exposed to E171 at 600 µg/g (*n* = 5); *p* = 0.827; Figure 1B) or the maternal weight gain per pup during gestation (2.6 ± 0.1 g/pup in the control group (*n* = 16) vs. 2.5 ± 0.2 g/pup in the exposed group (*n* = 5); Figure 1C). Regarding the morphological characteristics of the newborns, weight and craniocaudal length were measured daily until P7. The confidence interval for detecting pathological morphological development was defined based on 2 times the standard deviation from the mean values of the non-exposed animals. The results indicate that the growth of both the control animals and those exposed to E171 fell within this confidence interval (Figures 1D,E). All the data confirm that prenatal exposure to E171 by voluntary ingestion does not affect either the gestational parameters of pregnant mice (at least litter size and maternal weight gain) or the growth (weight and size) of their offspring.

### 3.2 E171 exposure during the gestational period alters breathing in neonates

**Figure 2:**
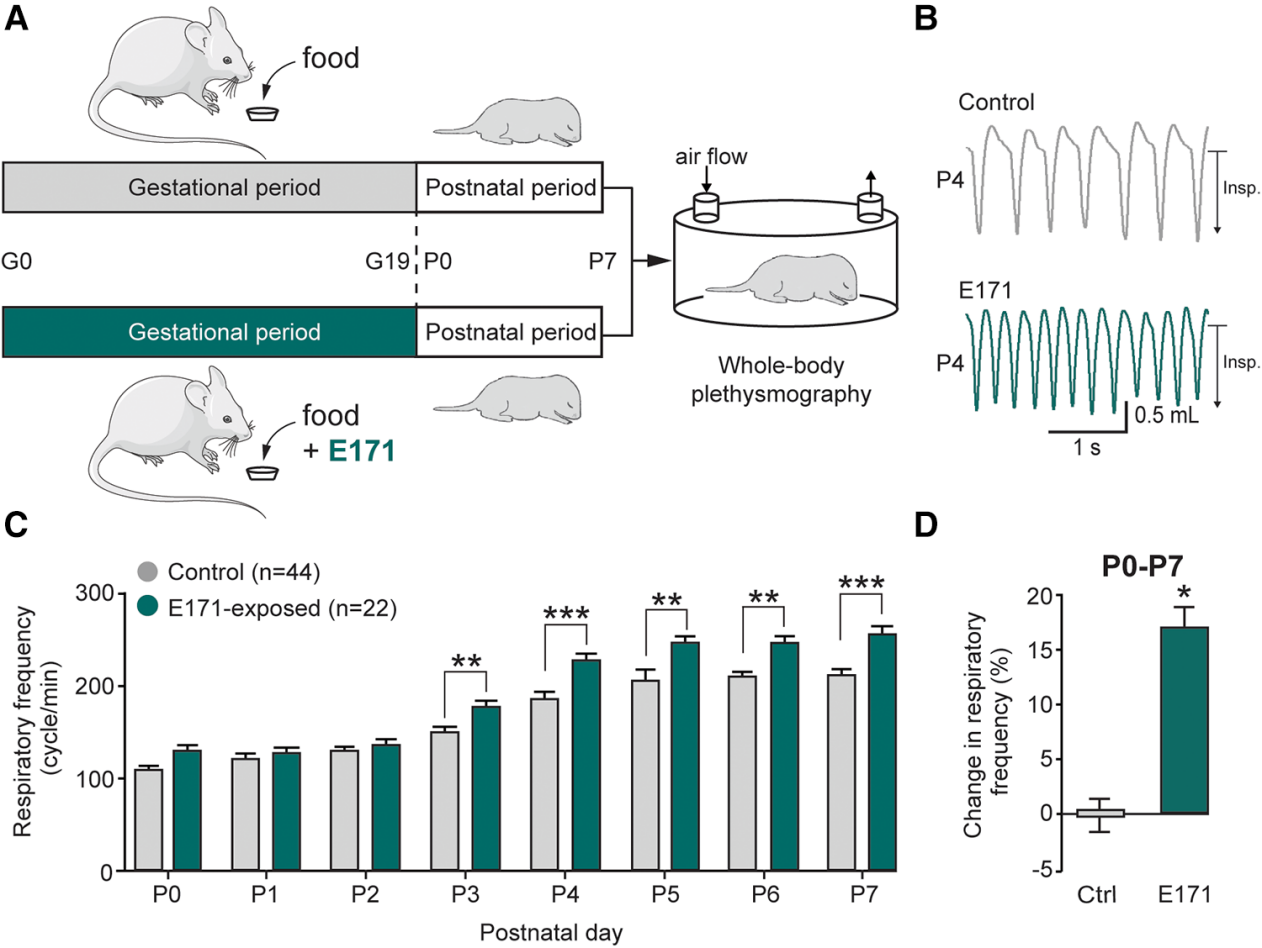
Breathing frequency is abnormally elevated during the first postnatal week in neonates that have been prenatally exposed to E171. (**A**) Schematics of protocols for non-exposed (grey) and E171-exposed (green) pregnant mice during the gestational (**G**) period and whole-body plethysmography experiments performs on offspring during the first postnatal (**P**) week. (**B**) Whole-body plethysmographic recordings of non-exposed control (upper trace, grey) and E171-exposed (lower trace, green) neonates obtained at P4. Insp., inspiration. (**C**) Bar chart (mean ± SEM) representing the evolution of breathing rate of neonates during the first postnatal week in control condition (grey bars) and after exposure to E171 during gestation (green bars). (**D**) Bar chart showing change in respiratory frequency (pooled data from P0 to P7) in non-exposed control (Ctrl, grey bar) and prenatally-exposed animals to E171 (green bar). *p < 0.05; **p < 0.01; ***p < 0.001. The mouse image is from Servier Medical Art website (smart.servier.com).

To assess the impact of chronic prenatal exposure to E171 on offspring breathing, whole-body plethysmography techniques were performed during the first postnatal week (Figure 2A). Similar to non-exposed neonates, the respiratory frequency in exposed neonates increased during the first seven postnatal days (106 ± 6 cycles/min at P0 and 205 ± 9 cycles/min at P7 for non-exposed neonates (*n* = 44) vs. 126 ± 9 cycles/min at P0 and 257 ± 7 cycles/min at P7 for exposed neonates at 600 µg/g of E171 (*n* = 22); Figure 2C). However, on the third postnatal day, the respiratory frequency of E171-exposed newborns during gestation was significantly higher than that recorded in non-exposed animals and remained elevated until P7 (176 ± 5 cycles/min (*n* = 22) vs. 150 ± 5 cycles/min (*n* = 44 at P3), respectively; *p* = 0.003; Figures 2B,C). During the first postnatal week, the respiratory frequency observed in E171-exposed animals increased by 17.2 ± 1.7% (*n* = 22; *p* < 0.001) compared to that observed in non-exposed animals (*n* = 44; Figure 2D).

**Figure 3:**
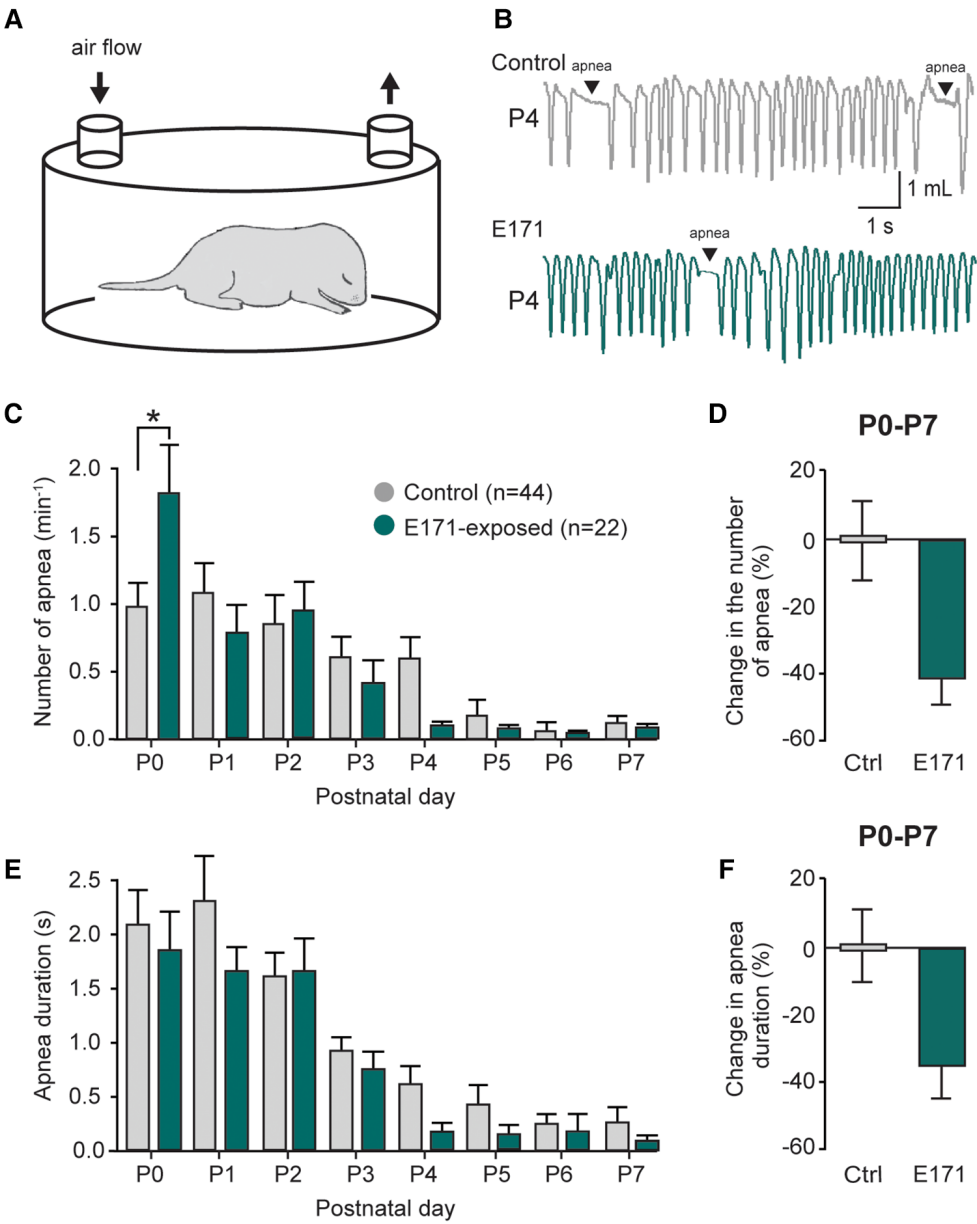
Changes in the number and duration of apneas in prenatally-exposed offspring. (**A**) Schematic of a plethysmography chamber. (**B**) Whole-body plethysmographic recordings of non-exposed control (upper trace, grey) and E171-exposed (lower trace, green) neonates obtained at P4. (**C**,**E**) Bar charts (mean ± SEM) representing the evolution of number **(C)** and duration (**E**) of apnea in newborns during the first postnatal week in control condition (grey bars) and after exposure to E171 during gestation (green bars). (**D**,**F**) Bar charts showing change in the number of apnea (**D**) and in apnea duration (**F**) in non-exposed control (Ctrl, grey bars) and prenatally-exposed animals to E171 (green bars). Data were pooled from P0 to P7. *p < 0.05.

Previously reported (11), The respiratory activity of neonatal mice is characterized by the presence of apnea during the first postnatal week. These events are a part of the normal development of respiratory function and are detected using the whole-body plethysmography technique (Figures 3A,B). In non-exposed newborns, these apneas were often observed during the first few postnatal days (1.10 ± 0.22 min^−1^ at P1; *n* = 44; Figure 3C, grey bars), and their occurrence gradually decreased during the first postnatal week (0.13 ± 0.06 min^−1^ at P7; Figure 3C). For the group exposed to 600 µg/g of E171 during gestation, we observed a similar trend over the first postnatal week, with a decrease in apnea frequency (0.79 ± 0.20 min^−1^ at P1 to 0.08 ± 0.05 min^−1^ at P7; *n* = 22; Figure 3C, green bars). Surprisingly and unexplained, we observed a significant elevation in apnea frequency in E171-exposed animals compared to the non-exposed group at P0 (1.86 ± 0.32 min^−1^, *n* = 22, vs. 0.98 ± 0.2 min^−1^, *n* = 44, respectively; *p* = 0.03; Figure 3C), while no difference was detected for the next six following days. However, when the data from P0 to P7 were grouped, it clearly showed a tendency toward reduced apnea frequency in the E171-exposed group (−42.25% ± 7.8%, *n* = 22; Figure 3D). Similarly to the apnea frequency, the apnea duration decreased during the first postnatal week in non-exposed animals (2.03 ± 0.35 s at P0 and 0.27 ± 0.13 s at P7; *n* = 44; Figure 3E) and in E171-exposed neonates (1.86 ± 0.32 s at P0 and 0.09 ± 0.05 s at P7; *n* = 22; Figure 3E). Regardless of the postnatal day, no differences were observed in these two groups, and the grouped data, while not statistically significant, indicated a tendency toward reduced apnea duration (−35.96% ± 9.41%, *n* = 22; *p* = 0.503; Figure 3F).

Importantly, these effects on breathing cannot be attributed to changes in pup morphology, as there were no significant differences in the weight and size of the prenatally exposed offspring compared to the control group (see Figures 1D,E). Taken together, these findings undeniably illustrate that prenatal exposure to 600 µg/g of E171 (by voluntary oral ingestion) impairs respiratory frequency in neonatal mice.

### 3.3 Maternal E171 exposure affects the neural regulation of their offspring's breathing

**Figure 4:**
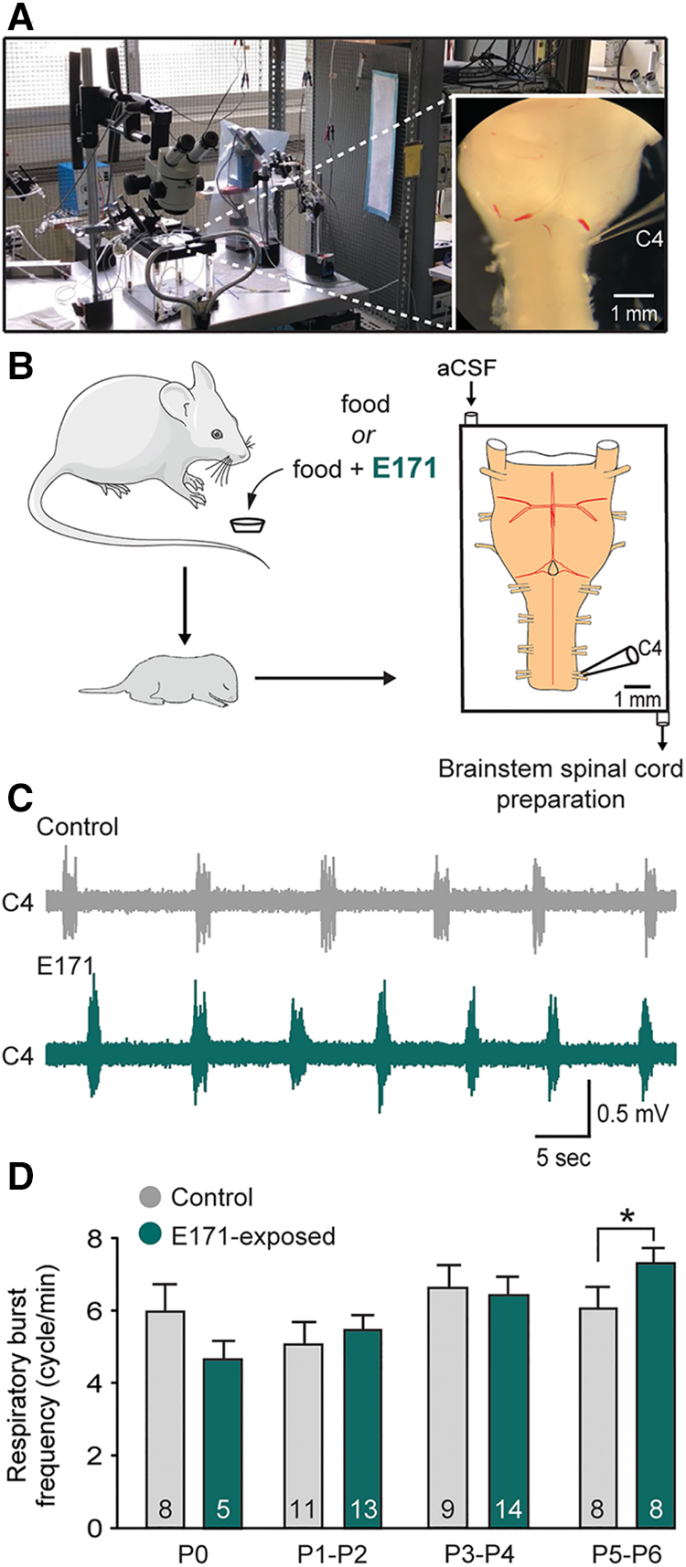
Effect of a chronic maternal exposure to E171 during gestation on respiratory-related motor burst activity generated in offspring *ex vivo*. (**A**) Photography of the experimental setup with a picture of a brainstem-spinal cord preparation (at right). (**B**) Schematics of maternal exposure to E171 and electrophysiological experiments performed on isolated brainstem-spinal cord preparations from offspring. aCSF, artificial cerebrospinal fluid. C4, 4th cervical ventral root. (**C**) Raw extracellular recording of spontaneous burst activity in a C4 ventral root of isolated preparations from unexposed (grey trace) and prenatally E171-exposed (green trace) neonates obtained at P5. (**D**) Bar chart showing the variation in spontaneous burst frequency (mean ± SEM) in non-exposed control (grey bars) and prenatally E171-exposed (green bars) groups during the first postnatal week. The number of animals is indicated in each bar. *p < 0.05. The mouse image is from Servier Medical Art website (smart.servier.com).

The previous results, indicating an abnormal elevation of the respiratory rhythm, could suggest an alteration in the central respiratory system characterized by an increase in the excitability of the respiratory neural centers. To test this hypothesis, we conducted electrophysiological recordings on isolated *ex vivo* brainstem-spinal cord preparations from offspring (ranging from P0 to P6; Figures 4A,B). These preparations contain the neural centers responsible for respiratory rhythm generation and spontaneously produce rhythmic respiratory-like activity, which we were able to record for extended periods at the cervical ventral root 4 (C4; Figures 4A–C), that carry phrenic motor axons to the diaphragm [the main inspiratory muscle; see also (15)]. Consistent with the previous *in vivo* findings, recordings of the respiratory-like rhythm in these isolated preparations from P0 to P4 showed no significant differences between the non-exposed and E171-exposed groups (6.64 ± 0.63 bursts/min (*n* = 9) vs. 6.42 ± 0.47 bursts/min (*n* = 14), respectively, at P3–P4; *p* = 0.778; Figure 4D). However, as similarly observed in *in vivo* experiments, there was a notable increase in the respiratory rhythm in the E171-exposed preparations compared to the non-exposed preparations on the 5th and 6th postnatal days (7.38 ± 0.29 bursts/min (*n* = 8) vs. 6.07 ± 0.62 bursts/min (*n* = 8), respectively; *p* = 0.01; Figures 4C,D). In conclusion, these electrophysiological findings align with the plethysmography results, and provide robust evidence that in mice, prolonged maternal exposure to 600 µg/g of E171 (by voluntary ingestion) can disrupt the fetal development of respiration. This disruption appears to be driven, at least in part, by modifications in the excitability of the central neural networks responsible for orchestrating the rhythm of this vital motor function.

## 4 Discussion

The objective of this study was to assess the potential toxicity of E171 on the developing respiratory centers in mice, and E171 was administered chronically during pregnancy through voluntary oral ingestion. We evaluated the respiratory function of the offspring *in vivo* using plethysmography, and recorded spontaneous respiratory-related activity *ex vivo* from brainstem-spinal cord preparations. Our findings indicate that in mice, prenatal exposure to E171 (600 µg/g) resulted in an abnormal increased breathing rate in neonatal mice starting from P3. Additionally, E171 had an excitatory effect on the spontaneously generated respiratory-related burst frequency in the isolated central nervous system from P5 to P6. It is worth noting that we assessed the morphological parameters of offspring during the first postnatal week due to previous findings that maternal exposure to TiO_2_ NPs can interfere with embryonic development. Indeed, it has been reported significant reductions in fetal weight gain, crown-rump length, and cauda length at E18 following chronic intragastric administration of TiO_2_ NPs at 100 µg/g (5). Consequently, these morphometric alterations could potentially affect aerobic metabolism, which might explain any changes in respiratory variables. However, in our study and similar to a prior investigation conducted with P25 TiO_2_ NPs (11), we did not observe any impact of prenatal E171 exposure on litter size, and neonates exhibited typical somatic growth. Thus, these differences may be attributed to the varying sizes of the TiO_2_ particles that were administered. In our study, the particles had a mean diameter of 115 nm, whereas the previous report used particles with a diameter of 6.5 nm (5). After inhalation or ingestion, it is widely accepted that characteristics of NPs (including the shape, size and crystalline phase) strongly influence their translocation in the body and potentially, their toxicity profile. Alternatively, the absence of observed morphometric alterations in our study could be related to differences in the method of NP administration. In our experiments, we employed voluntary ingestion, whereas the TiO_2_ particles were administered intragastrically in the previous study (6). Despite these discrepancies, it is reasonable to conclude that any changes in ventilatory parameters in our study were not a result of altered morphometric characteristics.

Recently, we demonstrate that a maternal exposure to TiO_2_ NPs (specifically, P25-type) during pregnancy affects the normal development and operation of the respiratory centers in progeny (11). However, P25-type NPs are primarily designed for technical applications like photocatalysis, metal coating, and semiconductor membranes, and while they can also be found in consumer products like cosmetics (e.g., sunscreens, lipsticks, toothpaste), the ingestion of P25 NPs is generally considered minimal. This contrasts with TiO_2_ E171, a common additive in food products, which is more likely to be ingested. Given that P25 and food-grade TiO_2_ E171 have been observed to exhibit different behaviors in environmental interactions (16), and thus may possess distinct toxicities (17), further research were required to assess the potential adverse effects of E171 on offspring's respiratory development. Similar to those obtained with P25 (11), our present findings clearly demonstrate that in mice, maternal exposure to 600 µg/g of E171 (by voluntary oral ingestion) alters specifically the respiratory rate of newborn offspring and exerts an abnormal excitatory influence on the central command of breathing. Our findings thus confirm prenatal toxicity of TiO_2_ (P25 (200 µg/g) and E171 (600 µg/g) by oral ingestion) to the developing respiratory function of neonatal mice.

The present study reveals a clear time course for the E171 (600 µg/g)-induced elevation in breathing rate, which differs from that observed with P25 (200 µg/g) as early as P0 in *in vivo* and *ex vivo* experiments (11). Indeed, although prenatal exposure to E171 resulted also in an abnormal increase in breathing rate, this effect only became evident from P3 *in vivo* and from P5 in *ex vivo* conditions. First, we can hypothesize that the distinct physicochemical properties of E171 and P25 nanoparticles (12, 13) may account for the different delay observed in the occurrence of respiratory effects. Nevertheless, another possible explanation is that, unlike P25, E171 NPs may have been retained within the organism, gradually released, and subsequently taken up by neural networks involved in the generation of respiratory rhythms. It is worth noting that food-grade TiO_2_ E171 is known to become entrapped by intestinal mucus *in vitro* when using intestinal epithelial cells (18). Interestingly, this phenomenon has recently been reported to be associated with the gradual accumulation of titanium in the lungs, observed seven days after a single intravenous injection of E171 (6 mg/kg body weight) in mice (19).

There is a growing evidence that exposure to food-grade E171 induces various adverse health effects (20), including alterations in the vasomotor (21) and cardiac functions (22), impairments of intestinal and systemic immune homeostasis (23, 24), ROS formation and genotoxicity (25, 26). However, very little is known about the potential toxic effect of E171 on the brain, especially during the vulnerable period of early development. We demonstrate here in mice that repeated maternal exposure to E171 (600 µg/g, voluntary oral ingestion) during pregnancy can alter the excitability of neurons involved in respiratory rhythmogenesis in newborn offspring. This may, in part, account for the abnormal increase in breathing rate observed in neonates a few days after birth. Future studies will be needed to determine whether the E171-induced elevation in breathing rate persists onwards. Indeed, some studies in the literature have reported long-term impairment of brain function following exposure to TiO_2_ NPs. For example, subcutaneous injections of TiO_2_ NPs in pregnant mice can lead to alterations in gene expression related to brain development. These changes include differences in the expression of genes associated with oxidative stress and those linked to neurotransmitters and psychiatric diseases in offspring (27). Administration of a single dose of TiO_2_ NPs (1 mg) by intravenous injection to female rats was sufficient to induce in the offspring behavioral deficits related to the autism spectrum disorder that persisted until the juvenile stage (8). Furthermore, long-term cognitive impairments have been reported in adult rats chronically exposed to 100 µg/g TiO_2_ NPs through *in utero* intragastric administration, with the effects persisting beyond P60 (7). Therefore, it is plausible that the E171-induced alteration of respiratory function could persist beyond P7, even after exposure has ceased at birth. As respiration is the primary process by which our bodies supply oxygen to cells, any dysfunction in respiratory activity may affect the growth and maturation of all brain cells. This could potentially contribute to neurodevelopmental disorders such as attention deficit, learning impairment, intellectual disability, or autism spectrum disorder.

## 5 Concluding remarks

To the best of our knowledge, this is the first study to demonstrate that *in utero*, exposure to E171 alone can impair postnatal respiratory function in mice. Our findings, which slightly differ from those obtained with P25 (11), highlight the importance of evaluating the toxicity of food-grade E171 rather than extrapolating results from P25 studies.

We assessed the respiratory function of neonatal mice from P0 to P7, which roughly corresponds to the first month and a half in human infants (28). Vulnerability to toxic compounds is higher during the perinatal period, and resultant developmental alterations can lead to long-lasting health issues. Considering that children are the primary consumers of E171-containing food products, such as sweets, assessing the perinatal toxicity of E171 is especially pertinent. Exposure to toxic substances during the critical embryonic period could potentially amplify individuals' sensitivity in later stages of life.

Finally, research on E171 toxicity plays a crucial role in the effort to determine an acceptable daily intake, which has been a challenging task thus far. These studies will inform risk managers on regulatory decisions regarding the use or withdrawal of this food additive. While nanoparticles hold immense potential across industries, their toxicity must be systematically assessed to guide policy-making for consumer safety.

## Data Availability

The original contributions presented in the study are included in the article/Supplementary Material, further inquiries can be directed to the corresponding author.
